# Neurocognitive function in children with cochlear implants and hearing aids: a systematic review

**DOI:** 10.3389/fnins.2023.1242949

**Published:** 2023-10-04

**Authors:** Jefferson Vilela da Silva Lima, Caroline Favaretto Martins de Morais, Nelma Ellen Zamberlan-Amorim, Patricia Pupin Mandrá, Ana Cláudia Mirândola Barbosa Reis

**Affiliations:** ^1^Postgraduate Program in Rehabilitation and Functional Performance, Ribeirão Preto Medical School, University of São Paulo, Ribeirão Preto, Brazil; ^2^Clinics Hospital of the Ribeirão Preto Medical School (HCFMRP-USP), University of São Paulo, Ribeirão Preto, Brazil; ^3^Department of Health Sciences, Ribeirão Preto Medical School, University of São Paulo, Ribeirão Preto, Brazil

**Keywords:** children, hearing loss, cochlear implants, hearing aids, cognition, speech perception, language

## Abstract

**Purpose:**

To systematically review the existing literature that examines the relationship between cognition, hearing, and language in children using cochlear implants and hearing aids.

**Method:**

The review has been registered in Prospero (Registration: CRD 42020203974). The review was based on the Preferred Reporting Items for Systematic Reviews and Meta-Analysis and examined the scientific literature in VHL, MEDLINE, CINAHL, Scopus, WOS, and Embase. It included original observational studies in children using hearing aids and/or cochlear implants who underwent cognitive and auditory and/or language tests. Data were extracted from the studies and their level of evidence was graded with the Oxford Center for Evidence-Based Medicine: Levels of Evidence. Meta-analysis could not be performed due to data heterogeneity. Outcomes are described in narrative and tables synthesis.

**Results:**

The systematic search and subsequent full-text evaluation identified 21 studies, conducted in 10 different countries. Altogether, their samples comprised 1,098 individuals, aged 0.16–12.6 years. The studies assessed the following cognitive domains: memory, nonverbal cognition, reasoning, attention, executive functions, language, perceptual-motor function, visuoconstructive ability, processing speed, and phonological processing/phonological memory. Children with hearing loss using cochlear implants and hearing aids scored significantly lower in many cognitive functions than normal hearing (NH) children. Neurocognitive functions were correlated with hearing and language outcomes.

**Conclusion:**

Many cognitive tools were used to assess cognitive function in children with hearing devices. Results suggest that children with cochlear implants and hearing aids have cognitive deficits; these outcomes are mainly correlated with vocabulary. This study highlights the need to understand children’s cognitive function and increase the knowledge of the relationship between cognition, language, and hearing in children using cochlear implants and hearing aids.

## 1. Introduction

Electronic hearing devices, such as hearing aids (HA) and cochlear implants (CI), can restore the experience of hearing to children with hearing loss. Interventions combining these technological resources with a period of appropriate speech-language-hearing rehabilitation not only enhance auditory input restoration but also promote greater development of auditory, cortical, language, and social skills (Sharma et al., 2002).

There is a consensus in the literature concerning the benefits of hearing devices to the development of auditory and language skills in children with prelingual hearing loss – especially with early interventions, which can enable them to have either a typical development or one near that of their hearing peers (Tanamati et al., 2011; Kral and Sharma, 2012; Kral et al., 2019). However, this favorable development scenario does not correspond to all cases. Hearing and language performances vary widely between individuals, which is not quite explained by the traditional predictive factors widely known in the literature (Geers et al., 2008; Niparko, 2010; Pisoni et al., 2010).

It has been recently proposed that neurocognitive functions and their development may be related to auditory and linguistic performance, influencing it in CI and HA users. Therefore, it is considered an underlying factor that could partly explain this population’s variable performance (Pisoni, 2000; Pisoni and Geers, 2000; Fitzpatrick, 2015).

Neurocognitive functions can be defined as a series of mental processes that involve knowledge acquisition, short-term memory (STM), long-term memory (LTM), working memory (WM), and operational memory, attention, perception, processing, reasoning, visualization, planning, problem-solving, and execution. These skills develop from the earliest years, progressing from the most basic to the most complex ones (Brandão et al., 2016).

Over the past few years, researchers have been engaged in exploring these neurocognitive functions’ roles and their relationship with hearing and language performances. The brain is a highly dynamic organ that depends on connections and experiences; hence, it is theorized that a period of auditory deprivation would have a substantial effect on both proximal areas (i.e., areas related to hearing) and distal areas, which would secondarily affect neurocognitive functions (Conway et al., 2009; Kral et al., 2016). However, some authors have considered the language deprivation hypothesis – i.e., the lack of language input (signed or spoken), rather than the lack of auditory input (Hall et al., 2017, 2018), may disrupt the development of neurocognitive skills.

Data from studies indicate that children using CI and HA perform significantly worse in various cognitive functions (Surowiecki et al., 2002; Harris et al., 2011). In line with those observations, studies have shown that children using HA and CI demonstrate deficits and/or indications of deficits in memory (Cleary et al., 2001; Burkholder and Pisoni, 2003; Lyxell et al., 2009), attention (Beer et al., 2014), phonological processing (Ambrose et al., 2012), and executive function (Charry-Sánchez et al., 2022). Additionally, findings by Kronenberger et al. (2014) suggest that hearing-impaired children are at two to five times greater risk of experiencing deficits in executive functions.

Furthermore, these measures are found to correlate with and be predictors of performance in speech perception measures (Beer et al., 2010; Ortmann et al., 2013; Ulanet et al., 2014; Castellanos et al., 2016). Therefore, neuropsychological assessment of hearing-impaired children may allow not only the identification of at-risk individuals (which would enable more targeted interventions) but also help understand how these neuropsychological processes work (as they are essential mechanisms in cognitive-linguistic processing and comprehension).

Given the above, this systematic review aimed to investigate whether there is a relationship between neurocognitive, auditory, and language skills in hearing-impaired children using hearing devices.

## 2. Materials and methods

The review was conducted according to the Preferred Reporting Items for Systematic Reviews and Meta-Analyses (PRISMA) (Page et al., 2021a).

### 2.1. Inclusion and exclusion criteria

Study eligibility criteria were defined according to the PICO acronym (population, interventions, comparators, and outcomes) (McKenzie et al., 2019). The review included studies in children (boys and/or girls), aged 0–12 years, with prelingual hearing loss, CI and/or HA users, submitted to cognitive, and auditory and/or language assessments. Studies involving populations with additional disabilities and auditory cognitive training were excluded.

Only primary studies were considered for inclusion, except for case reports. Secondary research, book chapters, annals, dissertations, theses, and animal models were excluded. Only studies published in Portuguese and English were reviewed, and no restrictions were applied regarding the year of publication.

### 2.2. Protocol registration

The review protocol was registered in the International Prospective Register of Systematic Review (PROSPERO) database under the registration number CRD42020203974.

### 2.3. Search strategy

Preliminary research was conducted to test, refine, and validate the strategy and terms adopted. The final strategy was reviewed by the authors.

The following databases were searched for potential studies: Virtual Health Library (VHL), Medical Literature Analysis and Retrieval System Online (MEDLINE/PubMed), Cumulative Index to Nursing and Allied Health Literature (CINAHL/EBSCOhost), Scopus, Web of Science (WOS), and EMBASE (Elsevier). CINAHL, WOS, and EMBASE were accessed via the CAPES/MEC Journal Portal.

The search terms were previously identified in Portuguese in the Health Science Descriptors (DeCS), and the corresponding vocabulary in English was identified in the Medical Subject Headings (MeSH). The controlled vocabulary in EMBASE (EMTREE) and CIHNAL (CINAHL Headings) was also consulted.

The strategies were modeled and adapted, when necessary, to ensure a highly sensitive search. All possible combinations were used between the following index terms, synonyms, and free terms: child preschool, child, hearing loss, deafness, cochlear implants, hearing aids, cognition, neuropsychological tests, executive function, memory, attention, auditory perception, speech perception, language, language development. During the research, the language filter was applied to retrieve only studies published in Portuguese and English. There was no limitation regarding the publication period, and retrieved studies had been published by August 2020.

### 2.4. Study selection

All records retrieved from the databases were imported into the reference manager EndNote Web to remove duplicates. The resulting records were exported to the Rayyan (web version) systematic review manager (Ouzzani et al., 2016) for screening.

Two reviewers (JV and NE) blindly and independently screened study titles and abstracts. Studies whose abstracts were unavailable were removed. Each record was labeled as “included,” “maybe,” or “excluded.” The reasons for excluding the latter were identified.

After such screening, the reviewers’ decisions were unblinded. When they diverged, the conflicting decisions were solved by a third independent reviewer (CF). Then, the selected records were imported to the Zotero reference manager for full-text reading. The CAPES/MEC Journal Portal was used as a primary alternative to retrieve full texts. If they were unavailable in this virtual library, a second attempt was made in ResearchGate. If this also failed, full texts were directly requested from the authors via ResearchGate.

After the retrieval, two reviewers (JV and CF) independently screened the full texts. If they diverged, it was solved through consensus and, if necessary, a third evaluator was consulted (ACR).

### 2.5. Data extraction

Two independent researchers (JV and CF) extracted data into a standardized Microsoft Excel spreadsheet to avoid measurement bias. Extracted data were compared, and any discrepancies were solved by discussion. The following data were extracted from each record: title, authors, year of publication, country of the study, study objective, sample size, participant exclusion and inclusion criteria, sex, chronological age, hearing deprivation time, age at the time of intervention, the device used, length of use, study design, and control group (if any). If the authors did not explain the research design, features of the design were noted.

Results were described regarding subjective and objective research instruments used for the audiological evaluation; speech, language, and cognitive functions; description of the cognitive domains evaluated; evaluation results; associations between tests; and study limitations.

### 2.6. Levels of evidence

Studies were evaluated according to the Oxford Center for Evidence-Based Medicine: Levels of Evidence (OCEBM Levels of Evidence Working Group, 2011). The levels of evidence were assessed by two independent judges; if they disagreed, a third judge solved the conflict.

### 2.7. Synthesis method

Due to outcome measure heterogeneity, it was not possible to conduct a meta-analysis. Therefore, the results were descriptively analyzed, and the summarized data are presented in tables.

## 3. Results

### 3.1. Search results

**Figure 1:**
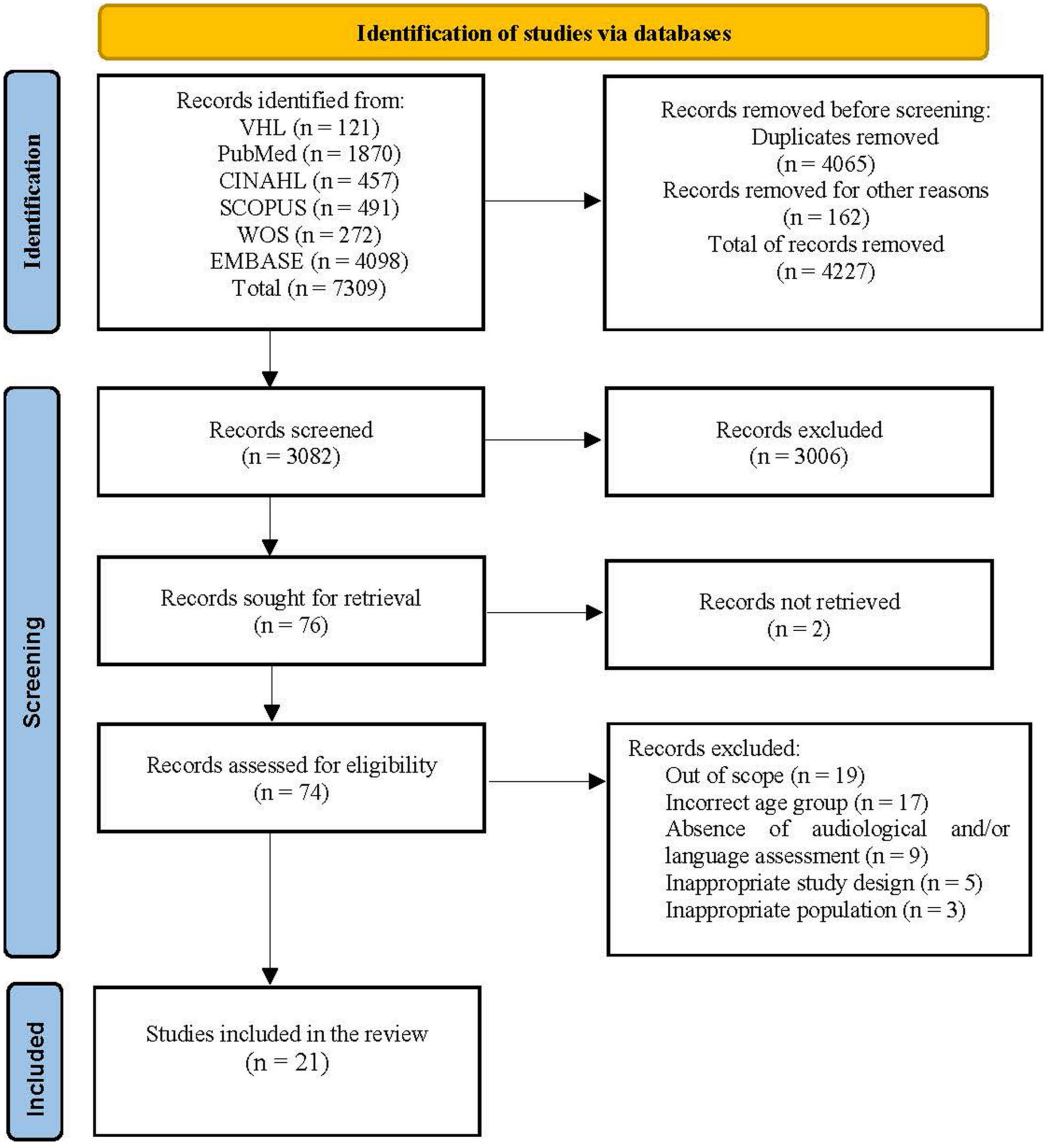
PRISMA 2020 flowchart depicting article identification and selection. CINAHL, Cumulative Index To Nursing And Allied Health Literature; VHL, Virtual Health Library; WOS, Web of Science.

The initial search identified 7,309 manuscripts. Duplicates were removed, totaling 4,065 records. Another 162 duplicates had not been identified at first and were manually removed. A total of 3,082 records were screened by title and abstract. Of these, 3,006 were excluded for not meeting the inclusion criteria. The full texts of 74 out of the 76 potential records were retrieved and screened for eligibility – 53 of them were excluded because they did not meet the inclusion criteria. Thus, 21 articles were included in the review. The study selection process with all PRISMA 2020 stages (Page et al., 2021a,b) is shown in Figure 1.

### 3.2. Characteristics of included studies

The general study characterization is provided in Table 1 and Supplementary Digital Content 1 (see Table, Supplementary Digital Content 1, which summarizes the characteristics regarding study design, objective, main outcomes, and level of evidence of the 21 studies included in the review). All 21 studies included in the review were published in English between 2002 and 2019. They were conducted in the following countries: the United States (Pisoni and Cleary, 2003; Conway et al., 2011a,b; Stiles et al., 2012; Beer et al., 2014; Bharadwaj et al., 2015; Meinzen-Derr et al., 2018; Davidson et al., 2019; McCreery et al., 2019), the United Kingdom (Figueras et al., 2008; Edwards and Anderson, 2014; Botting et al., 2017), Italy (Bubbico et al., 2007; Colletti et al., 2011), Australia (Dawson et al., 2002), Saudi Arabia (Hassan et al., 2014), South Korea (Lee et al., 2012), Denmark (Udholm et al., 2017), the Netherlands (de Hoog et al., 2016), Iran (Soleymani et al., 2016), and Taiwan (Lo and Chen, 2017).

**Table 1:** General characteristics of the studies included in the review, arranged by year of publication in ascending order.

Study number	Study title	Authors	Year of publication	Country
1	Short-term auditory memory in children using cochlear implants and its relevance to receptive language	DAWSON et al.	2002	Australia
2	Measures of working memory span and verbal rehearsal speed in deaf children after cochlear implantation	PISONI; CLEARY	2003	United States of America
3	Early hearing detection and intervention in children with prelingual deafness, effects on language development	BUBBICO et al.	2007	Italy
4	Executive function and language in deaf children	FIGUERAS; EDWARDS; LANGDON	2008	United Kingdom
5	Implicit sequence learning in deaf children with cochlear implants	CONWAY et al.	2011	United States of America
6	Infants versus older children fitted with cochlear implants: performance over 10 years	COLLETTI et al.	2011	Italy
7	Nonverbal cognition in deaf children following cochlear implantation: motor sequencing disturbances mediate language delays	CONWAY et al.	2011	United States of America
8	Phonological processing skills and its relevance to receptive vocabulary development in children with early cochlear implantation	LEE; YIM; SIM	2012	Republic of Korea
9	Vocabulary and working memory in children fit with hearing aids	STILES; MCGREGOR; BENTLER	2012	United States of America
10	Executive functioning skills in preschool-age children with cochlear implants	BEER et al.	2014	United States of America
11	Psycholinguistic abilities in cochlear implant and hearing-impaired children	HASSAN; ELDIN; AL KASABY	2014	Saudi Arabia
12	The association between visual, nonverbal cognitive abilities and speech, phonological processing, vocabulary and reading outcomes in children with cochlear implants	EDWARDS; ANDERSON	2014	United Kingdom
13	Working memory, short-term memory and reading proficiency in school-age children with cochlear implants	BHARADWAJ et al.	2015	United States of America
14	Auditory and verbal memory predictors of spoken language skills in children with cochlear implants	DE HOOG et al.	2016	Netherlands
15	Language skills and phonological awareness in children with cochlear implants and normal hearing	SOLEYMANI; MAHMOODABADI; NOURI	2016	Iran
16	Cognitive and outcome measures seem suboptimal in children with cochlear implants – a cross-sectional study	UDHOLM et al.	2017	Denmark
17	Nonverbal executive function is mediated by language: a study of deaf and hearing children	BOTTING et al.	2017	United Kingdom
18	Working memory capacity as a factor influencing the relationship between language outcome and rehabilitation in Mandarin-speaking preschoolers with congenital hearing impairment	LO; CHEN	2017	Taiwan
19	Language underperformance in young children who are deaf or hard-of-hearing: are the expectations too low?	MEINZEN-DERR et al.	2018	United States of America
20	Auditory, Cognitive, and Linguistic Factors Predict Speech Recognition in Adverse Listening Conditions for Children with Hearing Loss	MCCREERY et al.	2019	United States of America
21	Effects of early auditory deprivation on working memory and reasoning abilities in verbal and visuospatial domains for pediatric cochlear implant recipients	DAVIDSON et al.	2019	United States of America

### 3.3. Participant characteristics

The sample size of the studies ranged from 10 to 176 participants, totaling 1,098 hearing-impaired children who used CI and/or HA. Approximately 53% of the total population were males. Sex data were not available in four studies (Pisoni and Cleary, 2003; Figueras et al., 2008; Colletti et al., 2011; Conway et al., 2011b). Their mean chronological age ranged from 0.16 to 12.6 years, and the length of CI and/or HA use (hearing age) ranged from 0.04 to 8.7 years (see Table, Supplementary Digital Content 2, which presents the population characteristics). Ten out of the 21 articles did not mention the time of device use in their study populations.

Three studies included patients with mild to profound hearing loss (Botting et al., 2017; Lo and Chen, 2017; Meinzen-Derr et al., 2018), two included patients with mild to severe hearing loss (Stiles et al., 2012; McCreery et al., 2019), two included moderate to profound hearing loss (Bubbico et al., 2007; Figueras et al., 2008), three included severe to profound hearing loss (Hassan et al., 2014; Bharadwaj et al., 2015; Udholm et al., 2017), and 11 studies included patients with profound hearing loss (Dawson et al., 2002; Pisoni and Cleary, 2003; Colletti et al., 2011; Conway et al., 2011a,b; Lee et al., 2012; Beer et al., 2014; Edwards and Anderson, 2014; de Hoog et al., 2016; Soleymani et al., 2016; Davidson et al., 2019).

Regarding device use, 17 studies included participants who received unimodal auditory stimulation. Among them, four studies (Bubbico et al., 2007; Stiles et al., 2012; Lo and Chen, 2017; McCreery et al., 2019) specifically focused on HA users, all of them bilaterally. Eleven studies exclusively approached CI users (Dawson et al., 2002; Pisoni and Cleary, 2003; Colletti et al., 2011; Conway et al., 2011a,b; Lee et al., 2012; Beer et al., 2014; Edwards and Anderson, 2014; Bharadwaj et al., 2015; Soleymani et al., 2016; Udholm et al., 2017) and two studies included both groups (Figueras et al., 2008; Hassan et al., 2014). Additionally, three studies included participants with bilateral auditory stimulation (de Hoog et al., 2016; Botting et al., 2017; Davidson et al., 2019).

It is important to clarify that the studies by Conway et al. (2011a,b) included participants with bimodal stimulation and bilateral CI; however, they were tested with only one CI activated (the first CI). In addition, only one study (Meinzen-Derr et al., 2018) did not make it clear whether auditory stimulation was unimodal or bimodal and whether participants were unilateral or bilateral device users. Data on communication mode, therapeutic approach, and/or setting enrollment were absent in three (Bubbico et al., 2007; Hassan et al., 2014; Udholm et al., 2017) of the 21 studies. Hassan et al. (2014) reported that the children were attending regular language therapy sessions. However, the authors did not specify any details of the communication mode or therapeutic approach. Most of the study population (78.7%) used oral language as the main mode of communication, while 15.6% of participants used total communication (oral language associated with sign language). Only a small portion (5.3%) communicated exclusively through sign language.

The studies by Conway et al. (2011a,b) highlight that, although several of the children had been exposed to sign language, none of them relied exclusively on signs or gestures, and all children were tested using oral-only procedures. In addition, Botting et al. (2017) reported that part of their study population used Sign Supported English (an adapted sign system using English grammar) as their main communication mode. These participants were included in the group of exclusive sign language communication.

### 3.4. Cognitive measures

The 21 studies used heterogeneous neuropsychological tests/subtests, and some of them used more than one tool (see Table, Supplementary Digital Content 3, which outlines the cognitive tools and subtests used to assess the population and what domains were assessed). Altogether, 46 different cognitive tests/subtests were used to assess the following cognitive domains: STM (auditory and visual), WM (auditory, visual, and visuospatial), nonverbal cognition, reasoning, attention (auditory and visual), executive functions, language, perceptual-motor function, visuoconstructive ability, processing speed, and phonological processing/phonological memory. The Wechsler Intelligence Scale for Children (WISC) was the most frequently used test (47.6%), followed by NEPSY (28.6%) and Leiter International Performance Scale-Revised (19%).

#### 3.4.1. Memory and sequence learning

Several studies in this systematic review assessed memory, including both auditory/verbal and visual/visuospatial modalities.

Dawson et al. (2002) assessed STM performance in CI and NH children and found that NH children had superior performance in short-term memory tasks in both modalities evaluated. However, CI children did not seem to have a specific deficit in auditory STM. While this may be true, Bharadwaj et al. (2015) found differences in STM modality outcomes between children using CI and standard scores.

Hassan et al. (2014) also evaluated both STM modalities, but compared children using CI, children using HA, and NH children. Results showed that hearing impaired children had better visual STM than NH peers but did not have better results in auditory STM.

WM was also explored by some authors (Pisoni and Cleary, 2003; Conway et al., 2011a,b; Stiles et al., 2012; Beer et al., 2014; Edwards and Anderson, 2014; Hassan et al., 2014; Bharadwaj et al., 2015; de Hoog et al., 2016; Botting et al., 2017; Lo and Chen, 2017; Udholm et al., 2017; Davidson et al., 2019; McCreery et al., 2019). Results indicate that children using CI had an average performance in relation to the normalized sample in visuospatial/visual working memory and below average in auditory working memory (Edwards and Anderson, 2014; Bharadwaj et al., 2015). However, results suggest that children using CI have atypical sequence learning abilities with visual stimuli in comparison with NH children (Conway et al., 2011b). In contrast, Beer et al. (2014) did not find differences in the visual working memory of children using CI and their hearing peers. Also, the results found by McCreery et al. (2019) did not show any differences between HA and NH children. Shorter visual span results were also obtained with children using HA in comparison with NH children (Stiles et al., 2012).

Pisoni and Cleary (2003) obtained auditory short memory and auditory working memory capacity measures using forward and backward digit spans in children using CI and their NH peers. Children using CI had lower spans than NH children in both measures, with a greater difference in the forward digit spans. This points to a potential difference in the mechanisms used to encode and maintain the auditory working memory. Another study (Stiles et al., 2012) evaluated memory using forward and backward digit span with auditory and visual stimuli and found a significant effect regarding the stimuli modality for forward digit span performance outcome. Children had longer digit spans when the stimuli were presented via the auditory modality, which demonstrates that children using HA had a verbal coding preference. On the other hand, findings from Davidson et al. (2019) indicated that CI children tended to have significantly lower performance on simple and complex tasks that require verbal processing, such as verbal WM and fluid reasoning. However, they have similar performances in visuospatial processing tasks, suggesting working memory deficits in specific domains in children using CI.

De Hoog et al. (2016) compared children using CI with the normative sample in four STM measures and one WM measure. In all of them, children with CI scored significantly below the norm.

#### 3.4.2. Language, phonological and speed processing

Lee et al. (2012) and Soleymani et al. (2016) investigated the phonological processing (phonological awareness and phonological memory) skills of children using CI, and results showed that they scored significantly lower than NH children. Lower scores were observed even when children had received early implantation (Lee et al., 2012). Edwards and Anderson (2014) also revealed that CI children had performed worse in phonological processing than the mean score of the normalized sample.

#### 3.4.3. Attention, perception, motor function, and visuoconstructive abilities

Motor sequencing and tactile perception in children using CI were assessed by Conway et al. (2011a); the results showed that they performed at or near age-typical levels in tactile perception. On the other hand, they had significantly lower scores in fine motor sequencing measures than the normative values.

Attention is an important cognitive ability that allows one to select and focus on information that needs to be processed. Beer et al. (2014) investigated visual attention sustained in preschool children using CI and found that they scored lower than NH ones and the national norms. Concerning auditory attention, no significant differences were observed between children using HA and NH peers (McCreery et al., 2019).

As for visuoconstructive skills, a study by Conway et al. (2011a) showed that children using CI performed at age-appropriate levels, and Beer et al. (2014) did not find a significant difference between preschoolers using CI and their NH peers.

#### 3.4.4. Executive functions

Executive functions were evaluated in five studies (Figueras et al., 2008; Conway et al., 2011a; Stiles et al., 2012; Beer et al., 2014; Botting et al., 2017). Figueras et al. (2008) focused on comparing executive functions in children using HA or CI and hearing controls. Implanted and non-implanted children had significant performance differences in some areas of executive functions when compared with hearing peers, but did not differ significantly when the comparison was between HA and CI children. According to Conway et al. (2011a), children using CI presented an age-appropriate performance on the response inhibition task.

Botting et al. (2017) assessed different aspects of executive functions (executive-loaded visuospatial working memory, visuospatial cognitive fluency, cognitive shifting, executive planning, cognitive inhibitory control) in children with hearing impairment (HA and CI) and NH children. Children using CI and HA had lower scores than hearing peers in most executive function tests, except for visuospatial cognitive fluency.

Executive functions were also assessed with a parents’ survey. Results found by Stiles et al. (2012) did not find significant differences in the Planning and Sequential Processing LEAF subscale between children using HA and NH children. However, Beer et al. (2014) found that children with CI differed from NH children and the normative values in two out of three sub-scales of the BRIEF parent form. Children with CI had significantly more issues on the Inhibit and Working Memory subscales, though not on the Plan/Organize subscale, according to the caregivers.

### 3.5. Cognition and hearing

Study findings by Dawson et al. (2002) suggest that a period of auditory deprivation in early years did not cause a specific deficit in auditory STM. Likewise, findings by Pisoni and Cleary (2003) did not show a correlation between auditory digit span and hearing data, such as age at onset of deafness, duration of deafness, age at implantation, and duration of implant use. Furthermore, Bubbico et al. (2007) did not find a statistical difference between the early and late age of enrollment relating to nonverbal IQ. However, the lack of auditory input was associated with lower scores on the cognitive test.

In contrast, Conway et al. (2011b) established a positive correlation between sequence learning and hearing age (duration of implant use). In other words, the longer the children had auditory experiences through the implant, the higher were their scores. Beer et al. (2014) also established a relationship between the Planning/Organization BRIEF subscale and hearing age – the longer the duration of CI use, the fewer the problems with executive functions.

Regarding memory and speech perception tests, Pisoni and Cleary (2003) found a positive correlation between the performance in digit span and scores in spoken word recognition tests with forward digit span, explaining almost 7% of the variance in word recognition scores. Deficits in verbal WM seem to persist even in children with good audibility (Davidson et al., 2019).

### 3.6. Cognition and language

According to Dawson et al. (2002), STM performance accounts for significant variance in receptive language scores in children using CI, being spatial memory the strongest predictor. Bharadwaj et al. (2015) also reported a positive correlation between visual WM, visual STM, auditory STM, and reading measures. Opposite findings were stated by Davidson et al. (2019), only simple verbal WM tasks were significantly correlated with vocabulary scores. Thus, complex verbal WM and simple and complex visuospatial WM are not as closely related to language outcomes.

Similar results were reported by Hassan et al. (2014), who suggest that decreased auditory STM in children using CI and HA may be due to associated language impairments. Stiles et al. (2012) also found that children with poorer WM had a smaller vocabulary.

Pisoni and Cleary (2003) showed that early sensory and linguistic experiences immediately after cochlear implantation may have effects on the digit span. Children who were exposed to auditory/oral environments had longer forward digit spans than those in total communication environments; also, digit span was correlated with articulation rate measures. Conversely, another study did not find a significant correlation between digit span and articulation rate measures (Stiles et al., 2012).

Analogous results were obtained by de Hoog et al. (2016) concerning language input. Children enrolled in auditory/oral education performed better on lexical measures than those on total communication.

Findings by Lo and Chen (2017) suggest that language outcomes in Mandarin-speaking children using HA partially depend on their working memory capacity. Therefore, children with higher memory capacity performed as well as their hearing peers on the receptive and expressive language test.

An association between sequence learning abilities and standardized language measures was also reported by Conway et al. (2011b). Children with CI who performed better on sequence learning tasks also had better language outcomes. In another study, Conway et al. (2011a) found that motor sequence skills are closely associated with language outcomes in children using CI.

Language is a complex cognitive function. Lee et al. (2012) designed a study to investigate if metalinguistic skills could be a predictor of receptive vocabulary in children using CI. Results showed that phonological awareness was a significant predictor of vocabulary. Another study reported a relationship between phonological awareness and language skills in CI children, but not in NH children. In other words, language skills clearly predicted phonological awareness outcomes in children with CI (Soleymani et al., 2016).

Some studies sought a relationship between executive functions domain and language. Figueras et al. (2008) found a positive significant association between language ability and executive functions in children using CI and HA and in NH children. CI and HA children performed worse, especially when the test required language skills, suggesting that language and executive functions are interdependent but also dissociable. Therefore, the authors argued that executive function deficits are more likely to be a result of language delay caused by a lack of sensory auditory experiences rather than a period of deafness itself. Botting et al. (2017) investigated whether language mediates executive function differences in children using CI and HA and vice versa. The study findings showed that language not only influences executive functions but also plays a role in mediating their performance. Nevertheless, the reverse association may not happen; hence, poorer executive functions do not necessarily result in poorer language.

Beer et al. (2014) found a significant correlation with executive functions parent reports but not with their objective measures when data were controlled for language.

### 3.7. Hearing, cognition, and language

Colletti et al. (2011) measured the auditory, speech, and nonverbal cognitive function of children using CI implanted under 3 years old and later, in two distinct moments: at 5 years and 10 years post-implantation. Results showed that children implanted at earlier ages had superior performance in auditory, speech, language, and cognitive outcomes even after 10 years of CI use. On the other hand, results by de Hoog et al. (2016) showed that age at the onset of deafness and the first implantation did not correlate with measures of memory, language, and speech perception tests. Moreover, no correlation between memory and outcome performance on language measures was observed. However auditory perception tests contributed to a significant amount of variance in lexical and morphosyntactic language skills.

In the analysis by Edwards and Anderson (2014), age at implantation was a robust predictor of language, speech, and cognitive function performances of children using CI, and accounted for 7–15% of the variance across the outcomes. They also found that auditory memory accounted for 73% of the variability in measures of speech, phonological processing, vocabulary knowledge, and reading, and visual sequential reasoning and visual memory accounted for 16–25%.

No significant correlation was established between audiological data (including age at first HA, age at CI, and unaided pure-tone audiometry) and outcome measures such as verbal WM, visuospatial WM, perceptual fluid reasoning abilities, and receptive vocabulary (Davidson et al., 2019). On the other hand, Meinzen-Derr et al. (2018) created a language performance ratio that reflected language skills in relation to cognitive abilities. The study findings indicate that the degree of hearing loss and audibility (aided SRT) were factors associated with language underperformance. However, they did not find a significant association with the age at which children received the devices. Higher nonverbal IQ was significantly associated with higher language standard scores.

Udholm et al. (2017) investigated the relationship between cognitive skills, auditory capacity, speech perception, and intelligibility in CI children. They found that children whose cognitive performance was equivalent for their age achieved better results on auditory, speech, and language outcomes. Regardless of the years since implantation, CAP and SIR seem to reach a ceiling effect; hence, they are not the best tools to monitor this relationship between skills. The study concluded that vocabulary measured with PPVT-4 seems to reflect the effects of cognitive skills better than CAP and SIR.

McCreery et al. (2019) examined factors that could contribute to speech recognition in adverse listening conditions in children using HA. Their results showed that speech recognition was partially predicted by language, working memory, and auditory attention. In other words, children with better vocabulary and cognitive outcomes have better speech recognition in challenging conditions. Moreover, better aided speech audibility was a positive predictor of language.

## 4. Discussion

HAs enable most children experiencing hearing loss to hear and access speech and surrounding sounds through amplification. From another perspective, CIs restore certain aspects of hearing in children with severe to profound hearing loss who did not benefit from HAs, by providing direct stimulation to the auditory nerve through electrical signals. Despite their distinct characteristics and mechanisms, both have been employed as tools for early intervention in auditory habilitation for children with hearing loss, offering appropriate auditory stimulation, which prevents many detrimental effects of sensory deprivation. Hence, they can develop auditory and linguistic skills and learn alongside their NH peers. While hearing devices have a remarkable effect on children’s development, there is still a large variability in their outcomes that are not quite explained by conventional factors. The current review was designed to enhance our understanding of cognitive functions in children with CI and HA and how these functions interact with or influence their language and auditory abilities.

Many studies use cognitive tools to access children with hearing loss. Since this specific population is extremely heterogeneous, it is important to notice the number of different cognitive tools used to assess them in our review. This result highlights the absence of and the need for validated tests to assess this target population.

Memory was the most assessed domain. At least 71.4% of the studies assessed some aspect of memory, including auditory memory (Dawson et al., 2002; Pisoni and Cleary, 2003; Conway et al., 2011a,b; Stiles et al., 2012; Edwards and Anderson, 2014; Hassan et al., 2014; Bharadwaj et al., 2015; de Hoog et al., 2016; Lo and Chen, 2017; Udholm et al., 2017; Davidson et al., 2019) and visual/visuospatial memory (Dawson et al., 2002; Conway et al., 2011a,b; Stiles et al., 2012; Beer et al., 2014; Edwards and Anderson, 2014; Hassan et al., 2014; Bharadwaj et al., 2015; Botting et al., 2017; Davidson et al., 2019; McCreery et al., 2019).

There is no consensus regarding specific memory domain deficits. However, not surprisingly, several studies suggested that hearing-impaired children perform worse on auditory memory tasks and tend to perform on average or above average on visual memory tasks. When it comes to phonological awareness, children with CI tend to have lower ability levels and seem not to catch up with their hearing counterparts even when they receive early implantation. These results reinforce the critical role of auditory experiences in phonological awareness.

In addition, a period of deafness in early development may affect the executive functions’ building blocks. Findings with conventional executive function performance measures and parent report measures demonstrated that children with CI and HA have issues with some executive function domains, such as working memory (Figueras et al., 2008; Beer et al., 2014; Botting et al., 2017), shifting (Botting et al., 2017), planning (Figueras et al., 2008; Botting et al., 2017), inhibition (Figueras et al., 2008; Beer et al., 2014; Botting et al., 2017), impulse regulation (Figueras et al., 2008), cognitive flexibility (Figueras et al., 2008), and problem-solving (Figueras et al., 2008). However, some authors found age-appropriate performance in inhibition tasks (Conway et al., 2011a), cognitive fluency tasks (Botting et al., 2017), planning/organization subscale (Beer et al., 2014), and plan/sequential processing subscale (Stiles et al., 2012).

Overall results indicate that children with hearing loss have lower scores in many cognitive domains compared to NH peers.

Numerous studies have demonstrated close links between hearing, language, and cognitive outcomes. Relationships between hearing data and cognitive function were identified in some studies. Hearing age is associated with sequence memory learning (Conway et al., 2011b) and executive functions (Beer et al., 2014). Additionally, the age of intervention was a significant factor in faster improvements and a robust predictor of auditory, speech, language, and cognitive performances in children using CI (Colletti et al., 2011; Edwards and Anderson, 2014). Age-appropriate performance on cognitive measures has been associated with better performance in auditory, speech, and language measures (Udholm et al., 2017).

Comprehension in challenging environments requires cognitive resources. Language, working memory, and auditory attention have been found to explain speech recognition in children using HA (McCreery et al., 2019). This knowledge may enhance the importance of individual cognitive ability and how those abilities reflect significant variability in speech understanding.

Memory is a common underlying source that is involved in speech perception, language, and speech production. Findings suggest a significant association pattern between memory and speech perception (Pisoni and Cleary, 2003), receptive and expressive language (Dawson et al., 2002; Hassan et al., 2014; Lo and Chen, 2017), general language (Conway et al., 2011b), reading measures (Bharadwaj et al., 2015), vocabulary (Stiles et al., 2012; Davidson et al., 2019), and articulation measures (Pisoni and Cleary, 2003). Furthermore, motor sequencing was closely associated with language outcomes (Conway et al., 2011a).

Phonological awareness has been shown to predict vocabulary (Lee et al., 2012), demonstrating that it is a fundamental underlying skill for the development of language. Differences in phonological awareness performance among CI children can also be predicted by language skills (Soleymani et al., 2016).

Executive function is an umbrella term for a set of cognitive abilities that enable us to reason, solve problems, stay focused, have self-control, plan, and think (Cristofori et al., 2019). Results demonstrate that executive function is related to traditional language outcome measures in children using CI and HA (Figueras et al., 2008; Beer et al., 2014; Botting et al., 2017). Therefore, the findings raise the possibility that language may influence executive function, but the opposite may not be true (Botting et al., 2017).

## 5. Conclusion

Given the above, this systematic review suggests that cognitive functions can be related to hearing and language data. These results emphasize the wide range of assessment tools used to evaluate this specific population, particularly in terms of cognitive function. This provides an overview of the lack of consensus on instruments suitable for assessing CI and HA users. As a result, performance in language, cognitive, and hearing abilities is expected to vary.

Despite the variability, available evidence suggests that children with hearing loss perform worse in cognitive function than their hearing counterparts, which may account for variability in other outcomes. A better understanding of how these underlying mechanisms relate to auditory and language abilities can be useful for effective interventions. Our findings shed some light on the importance of individualized cognitive assessment in this specific population and the potential benefits that identifying cognitive risks or underperformance may bring to rehabilitation strategies and intervention monitoring.

Further research is required to understand whether specific intervention strategies based on their cognitive function may mitigate any effects on auditory and language ability function. In our study, only children without any other disability besides hearing impairment were included, as this factor could be a confounding variable. However, we acknowledge that including children with conditions that overlap or co-occur with hearing loss could help establish the direct and indirect impact on neuropsychological, language, and auditory development as well as their interaction. Future research should be oriented toward addressing these gaps.

## Data availability statement

The original contributions presented in the study are included in the article/Supplementary material, further inquiries can be directed to the corresponding author.

## Author contributions

JL contributed to study conceptualization and design, data collection, analysis, interpretation, article drafting and review, and final version approval. CM contributed to data analysis, article writing, and final version approval. NZ-A contributed to study design, data analysis, critical content review, and final version approval. PM contributed to data interpretation, critical content review, and final version approval. AR contributed to study conceptualization and design, data interpretation, critical content review, and final version approval. All authors contributed to the article and approved the submitted version.
