# Supplementary material for: Neurocognitive function in children with cochlear implants and hearing aids: a systematic review
Source: Front Neurosci. 2023 Oct 4;17:1242949. doi: 10.3389/fnins.2023.1242949 (PMC10582571; doi:10.3389/fnins.2023.1242949)
Supplement: Supplementary file 1 [file Data_Sheet_1.PDF]

Table 2. Characteristics of included articles regarding study design, objective, main outcomes, and level of evidence.

Study number	Study design	Objective	Results	OCEBM Levels of evidence
1.	Observational, analytical, case-control	To evaluate the performance of auditory sequence and short-term memory in children using CI and examine the relationship between these results and receptive language	CI users scored lower on auditory and visual STM tasks when compared to NHC and did not differ from their peers when stimuli were less likely to be verbally encoded. In addition, they do not appear to have deficits in sequential memory related to auditory modality when compared to NH. Performance on STM tasks significantly influenced the variability of results in the receptive language test, with visuospatial WM being the greatest predictor of this variation.	Level 4
2.	Observational, analytical, case-control	To investigate the relationship between digit span and speech perception	Children using CI present atypical development of STM, showing that there is a difference in relation to the mechanisms used to decode and maintain the sequence of digits in memory between CI children and NHC. Results showed a positive correlation between forward digit span and communication mode, children exposed to auditory/oral communication had greater span than those exposed to total communication. Furthermore, there was a positive correlation between the performance in the digit span and speech recognition tests, children with greater digit span had a higher percentage of word recognition, even after controlling for other variables. A strong correlation was also observed between speech rate and digit span. About 7% of the unexplained variation in speech recognition scores can be explained in terms of memory capacity.	Level 4
3.	Observational, analytical, cohort	To assess the cognitive and receptive language skills of children with prelingual HL in relation to the intervention age and verify the related variables	An inversely proportional relationship was observed between the mean PPVT score and the age of intervention. There was no correlation between the RSPM test and the intervention time (early and late). Time of hearing deprivation was associated with low scores on the cognitive test; however, the values are not indicative of impairment, and there was no significant difference between the early and late intervention groups. A positive correlation between language ability and degree of hearing loss was established.	Level 3
4.	Descriptive-comparative between groups	To compare the EF and language skills of implanted and non-implanted children with prelingual HL born into families of hearing parents with NHC and explore the association between language and EF in these groups	NHC showed better performance in the vocabulary and grammar assessment than children using CI and hearing aids. No differences were found in language outcomes between CI and hearing aid users. Performance differences were observed between the groups in some areas of EF. Compared to NHC, children using CI had lower performance on all EF measures, while those using HA had lower performance on some measures. A significant positive correlation was observed between the language score and the EF score for both NHC and HI children.	Level 4
5.	Observational, analytical, case-control	To assess what effects auditory deprivation and language delay may have on the general domain skills of sequential learning and investigate the possible role it plays in language outcomes after CI	Children using CI showed lower performance in visual sequence learning when compared to the NHC. Partial correlation between the time of CI use and the sequential learning ability; that is, children who experienced longer deprivation time obtained lower scores on the visual sequential learning test. There was an association between sequential learning ability and the results of standardized measures of language. This result seems not to be mediated by chronological age, WM, STM, or vocabulary. Overall, the results suggest that a period of auditory deprivation has secondary effects related to sequential learning skills, and these,	Level 4

			in turn, may partially explain the variability of language development in children with HL after cochlear implantation.	
6.	Observational, analytical, cohort	To investigate the effect of CI in terms of oral language skills and cognitive performance in children who received the device early versus those who received the device late	Children implanted before 12 months had faster gains and reached higher levels of auditory, speech, and language performance than children implanted after 12 months, this superior performance was maintained even after 10 years of follow-up. In addition, the early intervention group performed better on the cognitive nonverbal assessment (GMDS) and maintained higher scores on several LIPS-R subtests (nonverbal cognitive test) even after 10 years.	Level 2
7.	Observational, analytical, cross-sectional, case-control	To explore the effects of auditory deprivation on nonverbal cognitive skills and investigate whether individual variability in nonverbal cognitive skills may be related to post-CI language performance	The findings indicate that, although children with HI who use CI present age-normative performance levels in nonverbal cognitive skills related to visuospatial processing, visuomotor integration, and sensory discrimination, they seem to show impairment concerning fine motor sequencing. Furthermore, motor sequencing skills were closely associated with language outcomes in these children, suggesting that individual variability in sequencing functions is closely linked to language acquisition processes and therefore may help to explain the enormous variation in outcomes of speech and language and the benefit observed in this clinical population.	Level 4
8.	Observational, analytical, cross-sectional, case-control	To investigate the phonological processing skills in children using CI in relation to NHC and verify if these skills can explain the variability in the receptive vocabulary scores of children using CI	Children using CI had a significantly lower score in phonological awareness and pseudoword repetition tests compared to NHC. Among phonological processing skills, phonological awareness was a significant predictor for results on the receptive vocabulary test, suggesting that children with better performance in this skill have greater receptive vocabulary after implantation.	Level 4
9.	Observational, analytical, case-control	To determine whether children with mild to moderately severe sensorineural hearing loss have a WM deficit and whether there is a relationship between the deficit and receptive vocabulary size	Children using HA and NHC performed better in memory tasks for items presented by hearing than by the sight. There was no significant difference between the two groups regarding digit span and EF; however, HA children with EF deficits performed worse on the Corsi block task. The presence of background noise did not affect the performance in either group. HA users had a lower receptive vocabulary, and a significantly slower articulation rate when compared to NHC. There was a directly proportional positive correlation between vocabulary size, articulation rate, and performance in the auditory digit span task in the no-noise condition and the Corsi block task in the noisy condition.	Level 4
10.	Observational, analytical, case-control	To determine whether EF deficits occur in children using CI and if they can be identified at preschool age and verify if there is a relationship between EF and language skills, sociodemographic and auditory variables	Children using CI performed significantly worse in the visual attention task (concentration-inhibition) and WM compared to NH peers and the normative standard. No group differences were found in visual memory. Language correlated with EF measures; however, when data were controlled for language, differences in EF performance measures remained, while differences in EF problems reported by parents were no longer significant. Hearing age was the only auditory factor that had a statistically significant correlation with the BRIEF planning and organization scale.	Level 4

11.	Observational, analytical, case-control	To assess cognitive-linguistic skills in Arabic-speaking children using hearing aids or CI compared to NHC to estimate the nature and extent of any specific deficits in these children that could explain the different prognostic outcomes of the language intervention.	The results indicated that children using CI performed better in language and auditory STM than children using HA. In comparison to NHC, they performed worse in both tests, which may be associated with language impairment. However, in the visual STM and visuomotor skills tasks, children with HL performed better than the NHC.	Level 4
12.	Observational, analytical, cross-sectional, correlational	To establish whether there is a relationship between visual memory, visual reasoning ability, speech intelligibility, phonological processing, reading, and vocabulary in CI users and explore the relative strength of the associations between auditory and visual memory capacities and the results after CI	About half of the children reached levels below the normal range regarding auditory memory and receptive and expressive vocabulary. Significant and positive correlations were found between visual memory and reasoning tests and each of the outcome variables. A series of regression analyses revealed that for all outcome variables after accounting for variation attributable to the age of implantation, visual memory capacity and visual sequential reasoning ability together accounted for significantly greater variation (up to 25%) in each outcome measure.	Level 4
13.	Observational, analytical, cross-sectional	To investigate short-term and working memory through visual and auditory tasks in CI users and verify the relationship between these cognitive skills and reading and language measures.	Children using CI showed performance within the standard deviation compared to normative data in the visual STM and visual WM tests. However, they presented a below-average performance in the auditory STM, auditory WM, and verbal knowledge tasks. Concerning the reading measures, they obtained inferior performance in the comprehension measures, both oral and written (cloze), with a positive correlation being observed between these measures and those of STM and WM in the visual modality. Results showed a positive correlation between reading and auditory STM measures, except for the written comprehension (cloze) and fluency subtests, and auditory WM. Furthermore, a strong positive correlation was observed between measures of verbal and reading knowledge. No correlations were observed between performance on auditory WM.	Level 4
14.	Observational, analytical, cross-sectional	To verify if the variation in language results can be explained by auditory, auditory memory, and sociodemographic measures and analyze the degree of oral language delay in children with CI compared to NHC	Most children using CI did not achieve age-equivalent performance in lexical and morphosyntactic measures. Multiple linear regression measures revealed that lexical performance in children using CI was better predicted by age at the time of testing, phoneme perception, and auditory closure, while morphosyntax results were better predicted by lexicon, auditory closure, and auditory memory.	Level 4
15.	Observational, analytical, cross-sectional, case-control	To evaluate the language and metalinguistic skills of CI children compared to NHC and investigate the effect of auditory deprivation on the development of these children	The vast majority of CI children performed poorly on language measures when compared to NHC, and all had lower scores on the PA assessment, which suggests that PA tasks are sensitive to language experience. A significant correlation was observed between the measures of language and PA for the group of children using CI, but not for the group of NHC. The results also showed that language measures predict PA.	Level 4
16.	Observational, analytical, cross-sectional	To investigate whether cognition as a single variable significantly influences hearing, language, and speech outcomes in children with CI.	Children using CI who had cognitive performance within one standard deviation of the normal range to their age tended to achieve better hearing and speech scores than children below the equivalent age. Receptive vocabulary was significantly better in children with average cognitive scores. There was a	Level 4

			significant correlation between cognitive performance and the receptive language test. There seems to be a possible correlation between cognitive performance and auditory and speech development; however, it could not be demonstrated, due to the ceiling effect of the CAP and SIR scales. Thus, PPVT-4 seems to reflect the influence of cognitive abilities more adequately than CAP and SIR.	
17.	Observational, analytical, case-control	To investigate the EF and language of typically developing children with NH and children with HL and verify the existence of a correlation between these two skills and the possible influence that language exerts on EF, and vice versa	Children with HL using hearing devices performed significantly worse on EF tasks, except for the visuoconstructive skill task. There is a correction between language and EF, showing that language not only relates to EF but also has a role in mediating EF performance. However, an inverse association was not evident, suggesting that language is fundamental to EF performance but not the contrary.	Level 4
18.	Observational, analytical, case-control	To verify the influence of WM on the relationship between rehabilitation and performance in the receptive and expressive language in children with hearing impairment	The results of the present study support the idea that the WM capacity affects the performance of expressive and receptive language in children with HL. Children with HL and high memory capacity performed better than NH in receptive and expressive language. On the other hand, children with HL and low WM capacity had lower scores than NHC in receptive and expressive language. In addition, the rehabilitation time was positively correlated with the measure of expressive language among participants with high WM capacity. The data suggest that the language outcome of children with HL depends in part on the WM capacity.	Level 4
19.	Observational, analytical, cohort	To examine language performance in the context of cognitive skills in children with HL and identify factors associated with poor language performance (ratio of language skills relative to cognitive abilities)	41% of children with HL had a significant disparity between the language score and the nonverbal cognition score (low language performance). Children with low language performance were more likely to have higher nonverbal cognitive scores, although children with higher cognitive scores had higher average language performance. Factors associated with poor language performance included degree of hearing loss, aided hearing threshold, and socioeconomic status (maternal education and income level). A significant effect of unaided hearing threshold, degree of hearing loss, and audibility on the ratio of language in relation to cognitive abilities, as well as a significant effect on the chances of having inferior language performance.	Level 3
20.	Observational, analytical, case-control	To examine the influence of auditory, cognitive, and linguistic factors on speech recognition in adverse conditions for children with HL	Children with HL had worse speech recognition in noise and reverberation than NHC. Children with higher receptive vocabulary and WM skills had better speech recognition in noise and noise plus reverberation than peers with lower skills in these domains. Children with HL who had better aided hearing thresholds had better speech recognition in noise and reverberation than peers with lower thresholds. Better audibility thresholds were also associated with better language skills.	Level 4
21.	Observational, analytical, case-control	To compare verbal and visuospatial WM in NHC and CI children and verify if auditory deprivation affects WM and if those measures are related to receptive language level in CI children	CI children have domain-specific deficits related to the storage and processing of verbal information in WM. These deficits extend to receptive vocabulary and perceptual reasoning. That is, children with CI exhibited deficits in vocabulary and verbal WM compared to their NH peers and lower scores in the visual perceptual reasoning task when verbal coding was required for optimal performance. The deficit in verbal WM was maintained even with hearing thresholds lower than 30dBHL. Only simple verbal span was significantly	Level 4

	correlated with vocabulary scores. In contrast, they did not show deficits in their ability to store and process visuospatial information in WM and performed similarly to NHC. In addition, no differences were observed between the CI and NHC in the reasoning task that required purely visuospatial skills.
