# Supplementary material for: Neurocognitive function in children with cochlear implants and hearing aids: a systematic review
Source: Front Neurosci. 2023 Oct 4;17:1242949. doi: 10.3389/fnins.2023.1242949 (PMC10582571; doi:10.3389/fnins.2023.1242949)
Supplement: Supplementary file 2 [file Data_Sheet_2.PDF]

Table 3 – Population characteristics of the studies included in the systematic review

Study number	Sample								Hearing data		
	Participants	Eligibility		No	Age	B:G (%)	Control group		Duration of deafness	Age at first CI/HA	Length of CI/HA use
		Inclusion	Exclusion		Range or Mean (SD), yrs				Range or Mean (SD), yrs		
1.	Children using CI	Using 22-electrode CI, aged 5 to 11 years	--	24	5-11	50:50	NHC		0.66-6.58 (3.25)	1.5-6.58 (3.5)	0.5-8.5 (4.5)
2.	Children using CI	Aged 8 to 9 years, CI user for at least 3.5 years	--	176	8-9	--	NHC		--	--	--
3.	Children with congenital prelingual hearing impairment (early intervention and late intervention)	Bilateral prelingual sensorineural hearing impairment	Concomitant neurological syndromes.	70	5	58.6:41.4	--		--	--	--
4.	Children using CI and HA	Aged 8 to 12 years, prelingual sensorineural hearing impairment, born to hearing parents	Learning disabilities or significant development delay	47<br>CI: 22<br>HA: 25	8-12<br>CI: 9.8 (1.6)<br>HA: 10.8 (1.5)	--	NHC		--	CI: 3.5 (1.3)<br>HA: 2.0 (1.9)	CI: 6.4 (2.0)<br>HA: 8.7 (2.0)
5.	Children with profound prelingual hearing impairment	Aged 5 to 10 years, profound bilateral hearing loss onset at the age of 2, received CI by the age of 4, CI user for at least 3 years, and English as the first language	Known cognitive, motor, or sensory impairments; refused to participate in portions of the tasks and/ or displayed inattention or lack of motivation; performed more than 2SD from the mean in the Simon learning game	23	5.08-9.83<br>7.51 (1.66)	--	NHC		--	0.83-3.25<br>1.77 (0.69)	3-8.17<br>5.74 (1.62)
6.	Children using CI	Implanted under 3 years old; congenital deafness; no prior hearing experience, normal inner ears and cochleovestibular nerves; Nucleus CI 24M users	Meningitis as etiology, device failures; other known nonauditory disabilities	73	0.17-35<br>6.4	--	--		--	--	--
7.	Children using single CI	Prelingual, profound bilateral hearing impairment; received a CI by age 4; used CI for a minimum of 3 years, English as the first	Known cognitive, motor, or sensory impairments	24	5.08-9.83<br>7.5 (1.62)	62.5:37.5	NHC		--	0.83-3.25<br>1.75 (0.68)	3-8.17<br>5.76 (1.58)

		language, and hearing parents								
8.	Children using CI	Received a CI before the age of 24 months; aged 4 to 6 years, score over 85% in the open-set monosyllabic test; auditory/oral communication	Severe inner ear malformation and/or no additional disabilities (children with ASD, visual impairment, cognitive disabilities, etc.).	25	4.0-6.92<br>5.4 (0.79)	56:44	NHC	1.17 (0.61)	1.74 (0.32)	3.66 (0.86)
9.	Children with mild-to-severe sensorineural hearing loss fitted with bilateral HA	Oral communication; enrolled in an oral classroom; fitted with non-frequency compression HA	Visual impairment; behavioral difficulties	16	92.25	37.5:62.5	NHC	--	--	--
10.	Children using CI	Received CI prior to age 3; aged 3 to 6 years; onset of HL prior to age 36 months; profound hearing loss bilaterally, English as the first language spoken in a home environment; enrolled in aural rehabilitative program and/or educational setting; use of multichannel CIs	Any additional handicapping conditions other than hearing loss (children with ASD, and multiple handicaps)	24	3.13-6.94<br>4.36 (1.14)	58:42	NHC	0.08-2.5<br>0.72 (0.68)	0.87-3.05<br>1.67 (0.66)	0.53-5.18<br>2.73 (1.14)
11.	Children using HA or CI with delayed language development	Severe and/or severe-to-profound SNHL; HA/CI user for a minimum of 5 years.	Children with intellectual disability, below average mentality, any behavioral problems, pervasive developmental disorders, or auditory neuropathy	28	CI:<br>6-12.6<br>7.9 (0.9)<br>HA:<br>6-12<br>8.3 (1.3)	53.6:46.4	NHC	--	--	CI: 5 (1)<br>HA: 5 (2)
12.	Children using CI	Congenitally deaf or deafened before age 2 years; aged 5 to 12 years	Known significant developmental delay or neurodevelopmental disorders	66	5.4-11.7<br>8.5 (1.9)	32:34	--	--	1.2-8.9<br>3.7 (1.5)	1-10.1<br>5.13 (2)

13.	Children using CI	Prelingual, bilateral, severe-to-profound hearing loss; aged 7 to 11 years; auditory/oral communication; enrolled in mainstream educational settings	Developmental delays; vision problems, suspected of having or diagnosed with ASD; poor performance on the nonverbal test	10	7-11	60:40	--	--	--	--
14.	Children using CI	Prelingually, profound hearing impairment; using at least one CI	Developmental disorders; poor performance on the nonverbal test	39	5.3-10.1<br>8.0	56.4:43.6	--	--	0.58-3.92<br>1.75	3.1-8.6<br>6.2
15.	Children using CI	Farsi native speakers; profound congenital bilateral sensorineural hearing impairment; usage of Nucleus with 22 channels for at least 2 years; auditory/oral communication	Poor performance on the nonverbal test; structural or motor speech problems	18	5-5.5	38.9:61.1	NHC	--	--	--
16.	Children using CI	Severe-to-profound hearing impairment; implanted with Nucleus 24 device	--	58	2.8-16<br>9.4	50:50	--	--	0.6-16<br>3.5	0.04-11<br>3.4
17.	Children using CI and HA	Prelingual, sensorineural hearing loss; English or British Sign Language as the primary language	Explicit additional diagnoses such as global intellectual disability, autism, cerebral palsy, or Down syndrome	108<br>CI: 42<br>HA: 66	5.9-11.8<br>8.10 (1.9)	55:45	NHC	--	CI: 3.2 (1.9)	--
18.	Children using HÁ	Congenital, bilateral hearing impairment; enrolled in regular kindergarten classes; native Mandarin speakers; enrolled in auditory/oral rehabilitation approach	Multiple disabilities	39	5-6.1 (5.5)	53.9:46.1	NHC	--	--	--
19.	Children using CI or HA	Aged 0 to 6 years, bilateral hearing impairment of any degree	Unable to complete standardized testing; visual impairments, known syndrome or disorder that specifically affected language and	149<br>CI: 54<br>HA: 95	0.5-6.83<br>3.93 (1.57)	57.1:42.9	--	--	CI: 2.19 (1.23)	CI: 2.19<br>(1.41)

			primary language was not English							
20.	Children using HÁ	Mild to severe hearing impairment; aged 7 to 9 years; users of bilateral HA; English as the native language	Diagnosed developmental conditions	56	7.5 (0.6)<br>9.0 (0.4)	58.9:41.1	NHC	--	1.37	--
21.	Children using CI	Aged 5 to 9 years; spoken communication; hearing parents; enrolled in an auditory/oral education setting	Delays in cognitive development	25	5-9<br>7.9 (1.6)	58.3:41.7	NHC	1.4 (0.78)	2.5 (1.3)	--
