# Supplementary material for: Neurocognitive function in children with cochlear implants and hearing aids: a systematic review
Source: Front Neurosci. 2023 Oct 4;17:1242949. doi: 10.3389/fnins.2023.1242949 (PMC10582571; doi:10.3389/fnins.2023.1242949)
Supplement: Supplementary file 3 [file Data_Sheet_3.PDF]

Table 1. Summary of the assessment tools and domains evaluated in the studies included in this review

Study number	Audiological assessment		Cognitive assessment			Speech and/or Language assessment		
	Subjective	Objective	Assessment tool	Test/Subtest	Domain assessed	Assessment tool	Test/Subtest	Domain assessed
1.				Auditory tone-motor task	Auditory STM	CELF-P		Receptive language (vocabulary)
				Auditory word-imitation task				
				Auditory word-motor task				
				Visual hand-movement-imitation task	Visual STM	CELF-3		
				Picture-motor task				
			K-ABC	Hand movements	Nonverbal cognition (intelligence)			
				Triangles				
				Matrix Analogies				
				Spatial Memory				
				Photo Series				
2.	WIPI – closed-set word recognition		WISC-III	Forward digit span	Auditory STM	12 seven-syllable McGarr sentences		Speech intelligibility (articulation speed)
	k – open-set word recognition and lexical discrimination							
	BKB Sentence Test – open-set word recognition in sentence context			Backward digit span	Auditory WM			
3.	PTA		RSPM		Reasoning	PPVT		Receptive language (vocabulary)
4.			NEPSY	Tower Test	EF (Planning, problem-solving and self-monitoring, working memory, impulse regulation, and inhibition)	BPVS		Receptive language (vocabulary)
				Visual attention	Visual attention	TROG-2		Receptive grammar
				Design fluency	Visuospatial cognitive fluency			

				Knock and tap	EF (self-regulation and inhibition)			
				Day-Night and One-Two task	EF (inhibition)			
			D-KEFS	Card Sorting	EF (problem-solving, cognitive flexibility, and perseveration)			
5.			Magic Touch (Simon)		Visual sequence learning task (Visuospatial WM)	CELF-4	Concepts and following directions	General language ability
			WISC-III	Forward digit span	Auditory STM		Formulated sentences	
				Backward digit span	Auditory WM		Recalling sentences	
			CMS	Dot locations	Visuospatial learning and memory	PPVT-III		Receptive language (vocabulary)
6.	VRA or CPA	OAEs	GMDS	Locomotor	Non-verbal cognition function	PPVT - R		Receptive language (vocabulary)
				Eye and hand coordination and performance				
	CAP	ABR	Leiter-R	Figure-ground	Visuospatial attention	TROG		Receptive grammar
	IT-MAIS	Round window electrocochleography		Form completion				
		Electrically evoked round window ABR		Sequential order	Reasoning	SIR		Speech intelligibility
				Repeated patterns				
7.			WISC-III	Forward digit span	Auditory STM	PPVT-III		Receptive language (vocabulary)
				Backward digit span	Auditory WM			
			NEPSY	Fingertip tapping	Motor sequencing (nonverbal cognition)			
				Finger discrimination	Tactile perception (nonverbal cognition)	CELF- 4	Concepts and following directions	General language ability
				Knock and tap	EF (inhibition)			

				Design copy	Visual-motor integration and visuospatial processing		Formulated sentences	
			CMS	Dot locations	Visuospatial learning and memory		Recalling sentences	
8.			AKP	Elision, blending, and segmenting	Phonological processing (PA, lexical access, and PWM)	PPVT-K		Receptive language (vocabulary)
				RAN				
			Adapted version of Lee's study	Nonword repetition				
9.	PTA	REM	WISC-III	Block design	Perceptual Reasoning	PPVT-III		Receptive language (vocabulary)
				Picture Completion				
			LEAF scale	Planning and Sequential Processing	EF			
			McGarr Sentence Repetition (articulation rate)		Phonological loop			
				Forward digit span (auditory-quiet, auditory-noise, visual-quiet, and visual-noise)	Auditory and visual STM			
				Backward digit span (auditory-quiet, auditory-noise, visual-quiet, and visual-noise)	Auditory and visual WM			
				Corsi Span (quiet and noise)	Visuospatial WM			
			Adapted from O'Connor and Hermelin (1973)	Sequential encoding	Phonological loop or visuospatial sketchpad			
10.			NEPSY-II	Memory for design	Visual STM/WM	PLS-4		General language ability
			Leiter-R	Attention Sustained	Visual attention			
			Beery VMI		Visuoconstructive abilities			
			BRIEF/BRIEF-P	Inhibitory Control	EF			
				WM				
				Planning/Organizing				
			DAS-II	Picture similarities	Global nonverbal intelligence			

11.		ABR	ITPA: Arabic Version	Auditory sequential memory	Auditory STM	Arabic Language Test		Receptive and Expressive language
				Sound blending				
				Auditory closure				
		OAEs		Auditory association				
				Verbal expression				
				Visual sequential memory	Visual STM			
		Cochlear Microphonic		Visual reception				
				Visual closure				
				Manual expression				
12.			Leiter-R	Forward memory	Visual WM	CSIM		Speech intelligibility
				Sequential order	Reasoning			
				Repeated patterns		TOWK		Receptive and expressive language (vocabulary)
			CMS	Forward digit span	Auditory STM			
			NEPSY-II	Nonword repetition	Phonological WM	WIAT-II UK	Word reading	Reading
				Phonological processing	PA			
13.			WJ III COG NU	Auditory Working Memory	Auditory WM	WRMT-III	Word identification	Reading
				Numbers Reversed			Word attack	
			WISC-IV	Spatial Span Forward	Visuospatial STM		Word Comprehension	
				Spatial Span Backward	Visuospatial WM			
			KABC-II	Number Recall	Auditory STM		Passage Comprehension	
				Word Order				
				Hand Movements	Visual STM		Listening Comprehension	
				Verbal Knowledge	General knowledge			
				Riddles	Semantic memory (LTM)		Oral Reading Fluency	
				Expressive Vocabulary	Naming (LTM)			
14.	Dutch Nederlandse Vereniging Audiologen Woordlijsten – Phoneme perception		CPM		Non-verbal intelligence	T-TOS		Expressive and receptive language (vocabulary)
				Word span	Auditory STM			
	Auditory discrimination test			Memory sentences test				

	Word closure test		WISC-III: Dutch edition	Forward digit span				Morphosyntactic (expressive and receptive)
				Backward digit span	Auditory WM			
				Non-word repetition	Auditory STM			
15.	N/A	N/A	Developed by the authors	Syllabic awareness (syllable blending and segmentation)	PA (visually)	TOLD-P:3 – Farsi Version	Picture vocabulary	General language ability
				Intrasyllabic awareness (rhyme and alliteration detection)			Relational vocabulary	
							Oral vocabulary	
							Syntactic understanding	
				Phonemic awareness (detection of words with the same initial consonant, detection of words with the same final consonant, initial consonant detection, final consonant detection, initial consonant deletion, final consonant deletion, phoneme blending, and phoneme segmentation).			Sentence imitation	
							Morphological completion	
16.	CAP		Bayley-III	Cognition	Cognitive ability	PPVT-IV		Receptive language (vocabulary)
				Receptive communication	Language			
				Expressive communication				
				Fine motor skill	Motor ability			
				Gross motor skills				
			SON-R		Visual design/organization			
					Reasoning	SIR		Speech intelligibility
			WISC-IV		Language comprehension			
					Perceptual reasoning			

					Working memory		
					Processing speed		
17.				Odd-One-Out	Visuospatial WM	EOWPVT	Expressive language (vocabulary)
			WNV	Backward spatial span	Executive-loaded visuospatial WM		
			NEPSY	Design fluency	Visuospatial cognitive fluency		
			CCTT 1 and 2		EF (cognitive shifting)		
				ToL (computerized version)	EF (planning)		
				Simon Task (computerized version)	EF (inhibitory control)		
			WASI	Matrix Reasoning	Non-verbal cognitive ability		
			WISC-III	Symbol Search	Speed processing		
18.				Word span	Auditory WM	LDAP-R	Receptive and expressive language
19.	PTA		Leiter-R		Non-verbal cognitive ability	PLS-5	Receptive and expressive language
	SRT/SAT		MSEL	Visual reception			
20.	PTA	SII	AWMA	Odd-One-Out	Visuospatial WM	PPVT-IV	Receptive language (vocabulary)
	Aided sentence recognition (noise and noise + reverberation conditions)	REAR/ RECD	NEPSY-II	Auditory Attention	Sustained auditory attention		
21.	PTA			Visuoverbal forward digit span (computerized)	Simple Verbal Working Memory	PPVT-III	Receptive language (vocabulary)
				Counting Span (computerized)	Complex Verbal Working Memory		
	Open-set speech perception (LNT, CNC, or PKB)			Location Span (computerized)	Simple Visuospatial Working Memory		
				Odd-One-Out Location Span (computerized)	Complex Visuospatial Working Memory		

	WISC-IV	Matrix Reasoning<br>Picture Concepts	Perceptual reasoning (visual and visuospatial)

Abbreviations: STM – Short-term memory; CELF-P - Clinical Evaluation of Language Fundamentals – Preschool; K-ABC - Kaufman Assessment Battery for Children; WIPI - Word Intelligibility by Picture Identification Test; LNT - Lexical Neighborhood Test; BKB - Bamford-Kowal-Bench; WISC - Wechsler Intelligence Scale for Children; WM - Working memory; PTA – Pure Tone Audiometry; RSPM - Raven Standard Progressive Matrices Test; PPVT - Peabody Picture Vocabulary Test; BPVS - British Picture Vocabulary Scale; TROG - The Test for Reception of Grammar; D-KEFS - Delis-Kaplan Executive Function System; EF – Executive Function; CMS - Children's Memory Scale; CELF - Clinical Evaluation of Language Fundamentals; OAE - Otoacoustic Emissions; VRA - Visual Reinforcement Audiometry; CPA - Conditioned Play Audiometry; CAP - Category of Auditory Performance; ABR - Auditory Brainstem Response; GMDS - Griffiths Mental Developmental Scale; IT-MAIS - Infant-Toddler Meaningful Auditory Integration Scale; Leiter-R - Leiter International Performance Scale-Revised; SIR - Speech Intelligibility Rating; AKP - Assessment of Korean Preliteracy; RAN - Rapid Automatized Naming; PA - Phonological Awareness; PWM - Phonological Working Memory; LEAF - Learning, Executive, and Attentional Functioning; REM - Real-ear measures; Beery VMI - The Beery Developmental Test of Visual-Motor Integration; PLS - The Preschool Language Scale; BRIEF - The Behavior Rating Inventory of Executive Function; BRIEF-P - The Behavior Rating Inventory of Executive Function – Preschool Version; DAS - Differential Ability Scales; ITPA- Illinois Test of Psycholinguistic Abilities; CSIM - Children's Speech Intelligibility Measure; TOWK - Test of Word Knowledge; WIAT-II UK - Wechsler Individual Achievement Test-II UK; WJ III COG NU - Woodcock Johnson III Tests of Cognitive Abilities, Normative Update; WRMT-III – Woodcock Reading Mastery Test-III; LTM - Long-term Memory; CPM - Raven's Coloured Progressive Matrices; T-TOS - Test Instruments Developmental Language Disorders for Children; TOLD-P - Test of Language Development-Primary; Bayley-III - Bayley Scales of Infant Development; SON-R - Snijders-Oomen Nonverbal Intelligence Test Revised; WNV - Wechsler Non-verbal Scale of Ability; CCTT - Children's Color Trails Test; EOWPVT - Expressive One-Word Picture Vocabulary Test; ToL - Tower of London; WASI - Wechsler Abbreviated Scale of Intelligence; LDAP-R - Language Disorder Assessment for Preschooler-Revised version; SRT/SAT- Aided Speech Reception/Speech Awareness Thresholds; MSL - Mullen Scale of Early Learning; SII - Speech Intelligibility Index; AWMA - Automated Working Memory Assessment; RECD - Real-ear-to-coupler-difference; REAR - Real Ear Aided Response; PKB - Phonetically Balanced Kindergarten Wordlist; CNC - Consonant Nucleus Consonant
